# Monthly trend in mortality and length of stay among coronavirus disease 2019 (COVID-19) patients: Analysis of a nationwide multihospital US database

**DOI:** 10.1017/ice.2021.110

**Published:** 2021-03-19

**Authors:** Xiaozhou Fan, Barbara H. Johnson, Stephen S. Johnston, Nivesh Elangovanraaj, Prerna Kothari, Avrum Spira, Paul Coplan, Rahul Khanna

**Affiliations:** 1 Medical Device Epidemiology and Real-World Data Sciences, Johnson & Johnson, New Brunswick, New Jersey, United States; 2 MuSigma, Inc, Bangalore, India; 3 Lung Cancer Initiative, Johnson & Johnson, New Brunswick, New Jersey, United States

Since emerging in the first quarter of 2020, 33.6 million identified cases of coronavirus disease 2019 (COVID-19) caused by severe acute respiratory coronavirus virus 2 (SARS-CoV-2) have been reported, including >603,000 associated deaths in the United States as of mid-July, 2021.^1^ Recent evidence suggests that the case fatality rate has been declining among COVID-19 patients,^2,3^ with one study reporting at least a 34% decline over a 3-month period among all age groups.^2^ However, these studies have been restricted in their generalizability with samples limited to a single state^2^ or health system.^3^

## Methods

Using a nationwide, multihospital database, the Premier Healthcare Database (PHD),^4^ we identified patients with a diagnosis of COVID-19 (ICD-10-CM U07.1) admitted to an inpatient setting (ie, the first admission was considered the index admission). To be included, hospitals were required to provide continuous inpatient data from April 1, 2020 until July 31, 2020, to the PHD. We examined monthly trends (April 2020–July 2020) in mortality and length of stay (LOS) among the hospitalized patients. Then we assessed the relationship between admission month and study outcomes using a generalized estimating equations (GEE) model accounting for potential clustering of outcomes within hospitals, and adjusting for patient characteristics (age, gender, race, marital status, and payer), comorbidity status (Elixhauser comorbidity index score), provider characteristics (region, number of beds, location, and teaching hospital or not), and treatments for COVID-19 (admit to intensive care unit, use of ventilators, hydroxychloroquine, azithromycin, remdesivir, convalescent plasma, anticoagulants, dexamethasone, and methylprednisolone). Furthermore, we conducted a stratified GEE analysis according to age. Analyses were performed using R version 4.0.0 software (R Foundation for Statistical Computing, Vienna, Austria).

## Results

The final sample included 53,264 COVID-19 patients from 302 hospitals in 4 geographic regions: 118 (39.1%) were admitted in the South, 104 (34.4%) in the Midwest, 62 (20.5%) in the Northeast, and 18 (6.0%) in the West. Furthermore, 21,736 were admitted in April; 11,640 were admitted in May; 9,159 were admitted in June; and 10,729 were admitted in July. The distributions of mortality rate, LOS, and covariates included in the GEE model by admission months in the overall study cohort, in patients admitted to ICU, and in each age group are shown in Table 1. The mean age of the patients decreased from 64.1 years (±17.1) in April to 58.8 years (±19.2) in July (*P* trend < .0001). The mean Elixhauser comorbidity index score,^5^ used to asseses comorbidities among study patients, decreased from 3.7 (±2.3) in April to 3.2 (±2.2) in July (*P* trend < .0001), and the proportion of patients with an index score of 5 and above (indicating high underlying comorbidity burden) fell from 33.5% in April to 25.5% in July (*P* trend < .0001). The proportion of patients on mechanical ventilation decreased from 19.3% in April to 6.6% in July (*P* trend < .0001).

**Table 1.** Patient Characteristics, Treatment, and Outcomes and Provider Characteristics Among COVID-19 Hospitalized Patients in April, May, June, or July

Characteristics	April	May	June	July	*P* Value^a^
	No. (%)/Mean (SD)	No. (%)/Mean (SD)	No. (%)/Mean (SD)	No. (%)/Mean (SD)	
Total No.	21,736	11,640	9,159	10,729	
Deceased, No. (%)	4,543 (20.9)	1552 (13.3)	853 (9.3)	825 (7.7)	<.0001
LOS, d (mean)	9.37 (10.12)	9.02 (9.56)	7.47 (7.22)	5.30 (4.31)	<.0001
Age y (continuous)	64.13 (17.05)	61.02 (19.28)	58.04 (19.36)	58.84 (19.19)	<.0001
**Age y (categorical), no. (%)**					
<18	107 (0.5)	158 (1.4)	93 (1.0)	132 (1.2)	
18–39	1,966 (9.0)	1,629 (14.0)	1,765 (19.3)	1,824 (17.0)	<.0001
40–49	2,075 (9.5)	1,294 (11.1)	1,134 (12.4)	1,323 (12.3)	
50–59	3,645 (16.8)	1,906 (16.4)	1,565 (17.1)	1,811 (16.9)	
60–69	5,029 (23.1)	2,307 (19.8)	1,676 (18.3)	2,110 (19.7)	
70–79	4,319 (19.9)	2,063 (17.7)	1,519 (16.6)	1,851 (17.3)	
80+	4,595 (21.1)	2,283 (19.6)	1,407 (15.4)	1,678 (15.6)	
**Sex, no. (%)**					
Male	11,656 (53.6)	5,843 (50.2)	4,501 (49.1)	5,282 (49.2)	<.0001
Female	10,080 (46.4)	5,797 (49.8)	4,658 (50.9)	5,447 (50.8)	
**Race, no. (%)**					
White	8,649 (39.8)	5,429 (46.6)	4,730 (51.6)	6,091 (56.8)	<.0001
African American	5,017 (23.1)	2,573 (22.1)	2,165 (23.6)	2,644 (24.6)	
Other/Unknown	8,070 (37.1)	3,638 (31.3)	2,264 (24.7)	1,994 (18.6)	
**Payer, no. (%)**					
Commercial	4,735 (21.8)	2,327 (20.0)	2,251 (24.6)	2,739 (25.5)	<.0001
Medicare	11,589 (53.3)	5,740 (49.3)	3,944 (43.1)	4,854 (45.2)	
Medicaid	4,020 (18.5)	2,472 (21.2)	1,603 (17.5)	1,661 (15.5)	
Other	1392 (6.4)	1,101 (9.5)	1361 (14.9)	1,475 (13.7)	
**Marital status, no. (%)**					
Single	11,867 (54.6)	6,602 (56.7)	4,937 (53.9)	5,609 (52.3)	<.0001
Married	8,089 (37.2)	3,996 (34.3)	3,658 (39.9)	4,611 (43.0)	
Other/Missing	1,780 (8.2)	1,042 (9.0)	564 (6.2)	509 (4.7)	
Elixhauser score (continuous), no. (%)	3.71 (2.27)	3.71 (2.40)	3.34 (2.30)	3.20 (2.16)	<.0001
**Elixhauser score (categorical), no. (%)**					
0	1,308 (6.0)	957 (8.2)	916 (10.0)	1,067 (9.9)	<.0001
1–2	5,816 (26.8)	3,007 (25.8)	2,773 (30.3)	3,369 (31.4)	
3–4	7,336 (33.8)	3,621 (31.1)	2,836 (31.0)	3,559 (33.2)	
≥5	7,276 (33.5)	4,055 (34.8)	2,634 (28.8)	2,734 (25.5)	
**Hospital location, no. (%)**					
Urban	20,636 (94.9)	10,892 (93.6)	8,469 (92.5)	9,527 (88.8)	<.0001
Rural	1,100 (5.1)	748 (6.4)	690 (7.5)	1202 (11.2)	
**No. of beds**					
0–299	6,989 (32.2)	3,720 (32.0)	2,789 (30.5)	3,652 (34.0)	.0188
300–499	6,815 (31.4)	3,659 (31.4)	2,768 (30.2)	3,112 (29.0)	
≥500	7,932 (36.5)	4,261 (36.6)	3,602 (39.3)	3,965 (37.0)	
**Teaching hospital, no. (%)**					
No	8,103 (37.3)	5,270 (45.3)	4,722 (51.6)	5,971 (55.7)	<.0001
Yes	13,633 (62.7)	6,370 (54.7)	4,437 (48.4)	4,758 (44.3)	
**Provider region, no. (%)**					
Northeast	12,992 (59.8)	3,954 (34.0)	1,182 (12.9)	667 (6.2)	<.0001
Midwest	3,845 (17.7)	3,410 (29.3)	1,794 (19.6)	1,761 (16.4)	
West	515 (2.4)	311 (2.7)	505 (5.5)	611 (5.7)	
South	4,384 (20.2)	3,965 (34.1)	5,678 (62.0)	7,690 (71.7)	
Admission to ICU	5,139 (23.6)	2,966 (25.5)	2,214 (24.2)	2,006 (18.7)	<.0001
Mechanical ventilator	4,192 (19.3)	1,823 (15.7)	1,010 (11.0)	713 (6.6)	<.0001
Hydroxychloroquine	11,344 (52.2)	1,060 (9.1)	102 (1.1)	101 (0.9)	<.0001
Azithromycin	11,253 (51.8)	4,631 (39.8)	3,809 (41.6)	4,479 (41.7)	<.0001
Remdesivir	79 (0.4)	651 (5.6)	1,199 (13.1)	1,138 (10.6)	<.0001
convalescent plasma	339 (1.6)	359 (3.1)	285 (3.1)	250 (2.3)	<.0001
Anticoagulants	18,905 (87.0)	10,244 (88.0)	7,875 (86.0)	9,117 (85.0)	<.0001
Dexamethasone	1,032 (4.7)	958 (8.2)	3,143 (34.3)	6566 (61.2)	<.0001
Methylprednisolone	5,084 (23.4)	2,562 (22.0)	1,781 (19.4)	1,125 (10.5)	<.0001
**Patients admitted to ICU, no.**	5,139	2,966	2,214	2,006	
Deceased, no. (%)	2,095 (40.8)	976 (32.9)	605 (27.3)	545 (27.2)	<.0001
LOS, d (mean)	16.59 (14.20)	15.48 (12.75)	12.26 (9.29)	8.07 (5.66)	<.0001
**Patients Aged under 18, no.**	107	158	93	132	
Deceased, no. (%)	1 (0.9)	0 (0.0)	0 (0.0)	0 (0.0)	.1727
LOS, d (mean)	6.69 (9.35)	5.25 (7.65)	4.30 (4.05)	3.08 (2.75)	<.0001
**Patients aged 18–39 y, no.**	1,966	1,629	1,765	1,824	
Deceased, no. (%)	58 (3.0)	23 (1.4)	16 (0.9)	21 (1.2)	<.0001
LOS, d (mean)	6.26 (8.34)	5.66 (7.60)	4.55 (4.41)	3.66 (3.17)	<.0001
**Patients aged 40–49 y, no.**	2075	1,294	1,134	1,323	
Deceased, no. (%)	128 (6.2)	67 (5.2)	43 (3.8)	35 (2.6)	<.0001
LOS, d (mean)	8.41 (10.23)	8.13 (9.36)	6.68 (6.67)	4.83 (3.62)	<.0001
**Patients aged 50–59 y, no.**	3,645	1,906	1,565	1,811	
Deceased, no. (%)	429 (11.8)	174 (9.1)	103 (6.6)	67 (3.7)	<.0001
LOS, d (mean)	9.94 (11.46)	9.68 (10.23)	7.78 (7.78)	5.35 (4.30)	<.0001
**Patients aged 60–69 y, no.**	5,029	2,307	1676	2,110	
Deceased, no. (%)	967 (19.2)	360 (15.6)	176 (10.5)	177 (8.4)	<.0001
LOS, d (mean)	10.58 (10.91)	10.39 (10.38)	8.81 (7.94)	5.84 (4.60)	<.0001
**Patients aged 70–79 y, no.**	4,319	2063	1519	1851	
Deceased, no. (%)	1,289 (29.8)	411 (19.9)	232 (15.3)	222 (12.0)	<.0001
LOS, d (mean)	9.93 (9.76)	10.63 (10.03)	8.86 (7.77)	6.08 (4.78)	<.0001
**Patients aged ≥80 y, no.**	4595	2283	1407	1678	
Deceased, no. (%)	1,671 (36.4)	517 (22.6)	283 (20.1)	303 (18.1)	<.0001
LOS, d (mean)	8.88 (8.63)	8.79 (8.35)	8.57 (7.26)	6.00 (4.49)	<.0001

Note. COVID-19, the novel coronavirus 2; SD, standard deviation; LOS, length of stay; ICU, Intensive care unit.

a *P* values were based on Pearson’s correlation tests for linear trend for continuous, ordinary, and dichotomous variables; and *P* values were based on χ^2^ tests for nonordinary categorical variables (eg, race, payer, and marital status).

The mortality rate among hospitalized COVID-19 patients declined from 20.9% in April to 7.7% in July (*P* trend < .0001). The LOS also declined during this period, from 9.4 days (±10.1) in April to 5.3 days (±4.3) in July (*P* trend < .0001). Results from GEE analysis after accounting for the differences in the patient population by admission months indicate that COVID-19 patients admitted in May had 43% lower odds of mortality (odds ratio [OR], 0.57; 95% confidence interval [CI], 0.52–0.62), whereas those admitted in June had 56% lower odds of mortality (OR, 0.44; 95% CI, 0.39–0.49), and those admitted in July had 57% lower odds of mortality (OR, 0.43; 95% CI, 0.39–0.49) compared to patients admitted in April. Compared to the LOS among COVID-19 patients admitted in April, patients admitted in May had an average 4% shorter LOS (adjusted ratio of means, 0.96; 95% CI, 0.94–0.98), whereas patients admitted in June and July had 26% (adjusted ratio of means, 0.74; 95% CI, 0.72–0.76) and 46% (adjusted ratio of means, 0.54; 95% CI, 0.53–0.56) shorter LOS, respectively. The age-stratified GEE analysis revealed that the decrease in mortality was more pronounced in COVID-19 patients aged 60 years or older, and the decrease after June 2020 in LOS was consistent across all age groups. Among patients admitted to an intensive care unit (ICU), the mortality rates were 40.8% in April, 32.9% in May, 27.3% in June, and 27.2% in July (*P* trend < .0001). The LOSs for COVID-19 patients admitted to ICU were 16.6 days (±14.2) in April, 15.5 days (±12.8) in May, 12.3 days (±9.3) in June, and 8.1 days (±5.7) in July (*P* trend < .0001).

## Discussion

In the initial stages of the pandemic in the United States, the patients hospitalized with COVID-19 were mostly older individuals with high comorbidity burden,^6^ but as the pandemic has spread, an increasing number of hospitalizations have been reported among younger individuals.^7^ The decrease in average age and comorbidity status among COVID-19 cases as the pandemic has progressed has also been reflected in our sample of hospitalized patients. Reductions in mortality, as seen in this study, mirror those from other recent studies.^2,3^ We also observed significant reductions in LOS, especially in June and July (vs April); together with mortality reductions, these reductions suggest that the evolving clinical care for hospitalized COVID-19 patients is producing incremental gains in outcomes. The improvements in mortality and LOS were notable for COVID-19 patients admitted to ICUs, reflecting the incremental treatment gains among more severe cases. Several different medical and public health policy factors may have contributed to these meaningful declines in mortality and LOS: improved clinical experience among physicians, better hospital protocols in patient management, more effective treatment options, increased usage of masks and social distancing measures reducing the volume of hospitalization pressure on healthcare systems.^8^
